# A gateway towards non-collinear spin processing using three-atom magnets with strong substrate coupling

**DOI:** 10.1038/s41467-017-00506-7

**Published:** 2017-09-21

**Authors:** J. Hermenau, J. Ibañez-Azpiroz, Chr. Hübner, A. Sonntag, B. Baxevanis, K. T. Ton, M. Steinbrecher, A. A. Khajetoorians, M. dos Santos Dias, S. Blügel, R. Wiesendanger, S. Lounis, J. Wiebe

**Affiliations:** 1 Department of Physics, Hamburg University, 20355 Hamburg, Germany; 2 Peter Grünberg Institute and Institute for Advanced Simulation, Forschungszentrum Jülich & JARA, Jülich, 52425 Germany; 3 Leiden Institute of Physics, Leiden University, 2333 CA Leiden, The Netherlands; 4 Institute for Molecules and Materials (IMM), Radboud University, 6525 AJ Nijmegen, The Netherlands

## Abstract

A cluster of a few magnetic atoms on the surface of a nonmagnetic substrate is one suitable realization of a bit for spin-based information technology. The prevalent approach to achieve magnetic stability is decoupling the cluster spin from substrate conduction electrons in order to suppress destabilizing spin-flips. However, this route entails less flexibility in tailoring the coupling between the bits needed for spin-processing. Here, we use a spin-resolved scanning tunneling microscope to write, read, and store spin information for hours in clusters of three atoms strongly coupled to a substrate featuring a cloud of non-collinearly polarized host atoms, a so-called non-collinear giant moment cluster. The giant moment cluster can be driven into a Kondo screened state by simply moving one of its atoms to a different site. Using the exceptional atomic tunability of the non-collinear substrate mediated Dzyaloshinskii–Moriya interaction, we propose a logical scheme for a four-state memory.

## Abstract (web summary)

Information technology based on few atom magnets requires both long spin-energy relaxation times and flexible inter-bit coupling. Here, the authors show routes to manipulate information in three-atom clusters strongly coupled to substrate electrons by exploiting Dzyaloshinskii–Moriya interactions.

## Introduction

Information technology is currently changing the paradigm from the separate use of the charge and spin degrees of freedom of the electrons toward a combination of both properties in so-called spin-electronic elements^1^. Using the scanning tunneling microscope as a tool, arrays of a well-defined number of atoms and shape can be assembled on a nonmagnetic substrate^2^, and their spin state^3^ and excitations^4^ can be read out atom-by-atom. Recently, corresponding arrays consisting of magnetic atoms have been intensely studied as the basic constituents for future spin-based information storage^5, 6^ and processing^7^ schemes.

One of the main hurdles in the former respect is to achieve long spin-energy relaxation and -decoherence times^8, 9^ of the spin states, which is usually established by two ingredients. First, a large energy barrier separating the spin states, provided by the so-called magnetic anisotropy energy (MAE)^10^, is needed, in order to prevent the system from undergoing thermally induced fluctuations at sufficiently low temperatures. Second, the spin states need to be protected from quantum tunneling^11^, and from scattering by electrons (the so-called Kondo scattering^12^) or by phonons^13^ from the substrate, which can shortcut the MAE barrier. To this end, an appropriate tuning of the MAE^14^ and a decoupling of the spins of the array from the substrate electrons using rare earth atoms^15, 16^ in combination with thin decoupling layers^13, 17, 18^, superconducting^19^, or semiconducting substrates^20^, was usually pursued. However, with respect to spin processing, a tunable communication between the array-spins is essential. Following the decoupling layer approach, communication with stable spin states is so far restricted to dipolar and superexchange coupling which offers limited flexibility.

A much larger flexibility can be achieved by the contrary, i.e., strongly coupling the array-spin to substrate electrons leading to Ruderman–Kittel–Kasuya–Yosida (RKKY) interactions^21^, which can transfer spin information between two arrays^7^, and can be tuned in strength, sign and even non-collinearity^22^. In view of applications, it is, therefore, a formidable task to realize an array of few atoms with a long spin-energy relaxation time useable for information storage, which at the same time, is sufficiently coupled to substrate conduction electrons in order enable strong RKKY coupling to the array. Moreover, the use of heavy substrate materials would additionally feature strong spin-orbit coupling, leading to highly non-collinear Dzyaloshinskii–Moriya contributions to the RKKY interaction^22^, which can potentially add functionality to the system.

Long-range interactions are typically promoted in a substrate material that almost fulfills the Stoner criterion for ferromagnetism, such as Rh^23^, Pd^24^, or Pt^10, 21, 22, 25–28^. As a consequence, such materials display a large cloud of magnetically polarized host atoms, leading to a strongly enhanced total magnetic moment of the impurity-polarization cloud system, which was, therefore, referred to as a giant moment (GM) in the early days of research on magnetic alloys^29^. Theses heavy substrate materials also support non-collinear magnetization states of the GM systems and Dzyaloshinskii–Moriya interactions^22^, and are thus ideal candidates for the pursuit of a magnetically stable cluster, which can be accessed by strong non-collinear RKKY interactions. With respect to information storage and processing applications, the main open questions are, thus, whether the MAE of such a non-collinear GM system can be tuned such that Kondo screening^30^ and quantum tunneling processes^13^ are avoided and the system is driven to support stable spin states as in a classical magnet^31^.

In order to realize a system that encompasses all these requirements, we built giant moment clusters (GMCs), each consisting of only three iron (Fe) atoms that are assembled into a triangular array on the (111) surface of a platinum (Pt) single crystal. We show that we can indeed store information for hours in the non-collinear spin state of such a GMC, enabling a tuneable non-collinear interaction between atomic scale bits for flexible schemes of information processing.

## Results

### GMC fabrication

**Fig. 1:**
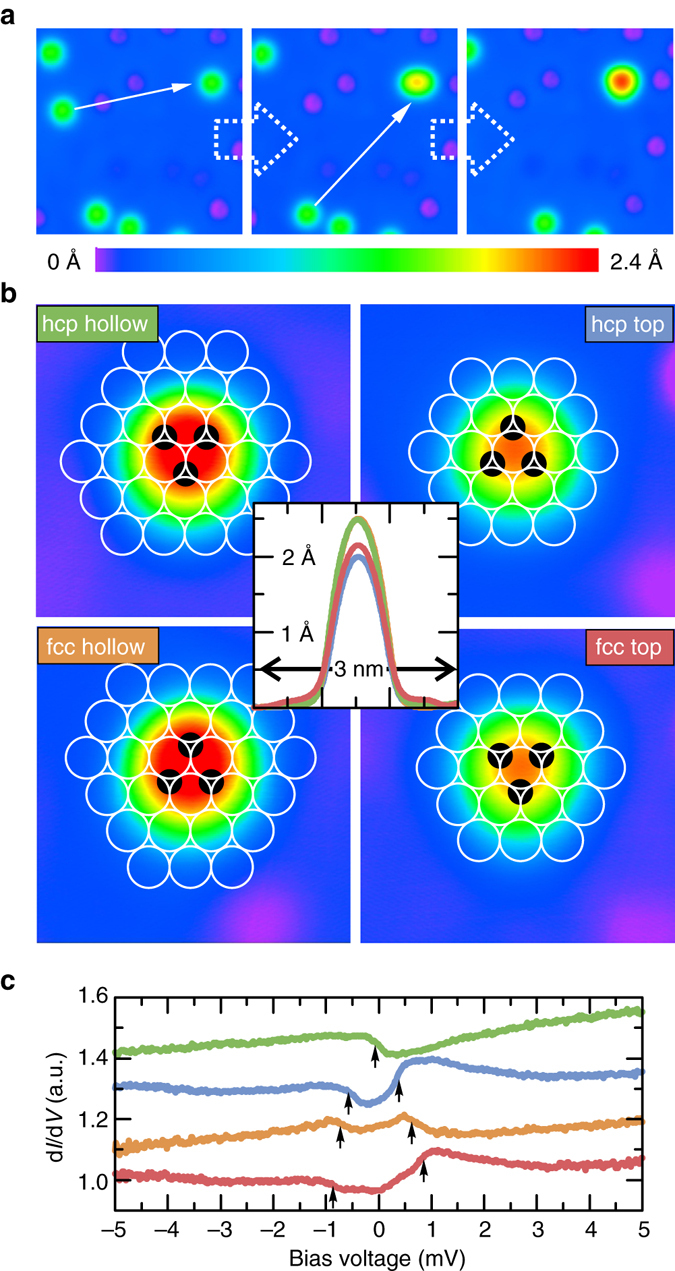
Fabrication of the giant moment clusters. **a** Constant-current images taken during the fabrication of an Fe_3_ cluster from three individual Fe atoms on Pt(111) (image sizes 5×5 nm^2^, *V* = −5 mV, *I* = 500 pA). *Left*: the *arrow* indicates the first manipulation step of one of the individual Fe atoms; *Middle*: a dimer has been formed, the *arrow* indicates the second manipulation step; *Right*: the Fe_3_ cluster has been formed. **b** Constant-current images of the four possible Fe_3_ cluster geometries (image sizes: 2×2 nm^2^, *V* = 5 mV, *I* = 500 pA). The *open circles* indicate the underlying lattice of surface Pt atoms and the *black spheres* the positions of the three Fe atoms within each cluster. A height profile across the center of each of the individual clusters is indicated in the middle. **c** ISTS taken on the center of the hcp hollow (*green*), the hcp top (*blue*), the fcc hollow (*orange*) and the fcc top (*red*) cluster (*B* = 0T, *T* = 0.3 K). The step-like features in each spectrum originating from the excitations of the clusters are indicated by *arrows*

The assembly of the GMCs is shown in Fig. 1 (see Methods). Each Fe atom can sit on one of the two hollow adsorption sites, face centered cubic (fcc) or hexagonally closed packed (hcp), on the lattice of the Pt surface atoms^32^. The Fe atoms are free from adsorbed hydrogen^33^ as indicated by their small apparent height (see Supplementary Fig. 1). By lateral atom manipulation three Fe atoms are placed artificially on next nearest neighboring adsorption sites of the same type in the most compact triangular geometry (Fig. 1a). In this way, four different species of GMCs can be built (Fig. 1b, see Supplementary Note 1 and Supplementary Fig. 1 for the unambiguous determination of the stacking). For each of the two adsorption sites, fcc and hcp, the cluster can be either positioned such that a Pt atom is underneath the center of the trimer, referred to as top, or such that a hollow site is underneath the center, referred to as hollow. The two hollow site trimers have a larger apparent height (≈250 pm) in comparison to the top site trimers (≈200 pm). The electronic properties of all four trimer types are characterized by (inelastic) scanning tunneling spectroscopy ((I)STS, see Methods) as shown in Fig. 1c. Their spectra show distinct excitations (arrows), which are markedly different from the excitations of isolated Fe atoms outside of trimers^32^, indicating the emergence of a coherent electron state after the formation of the trimer.

### Density functional theory calculations

**Fig. 2:**
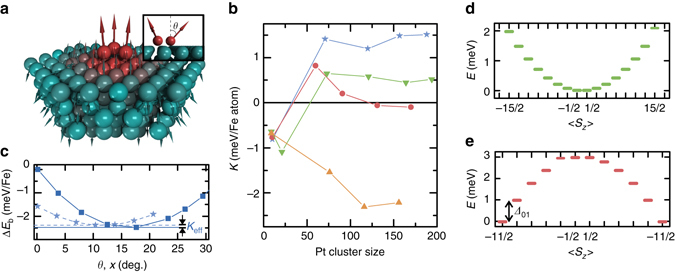
Magnetic anisotropy and energy level schematics of the giant moment clusters. **a** Model of the hcp-top Fe_3_ cluster on the Pt surface resulting from the ab-initio (KKR) calculations indicating the strong non-collinearity of the magnetic moments (*arrows*) in the cluster and the polarization (*color*) in the Pt. The *inset* shows the definition of the non-collinearity angle *θ*. **b** Collinear ab-initio calculated MAE of the hcp hollow (*green*), the hcp top (*blue*), the fcc hollow (*orange*), and the fcc top (*red*) cluster geometries as a function of the number of considered Pt atoms. **c** DFT non-collinear band-energy calculations (energy per Fe atom) of the hcp top cluster as a function of the non-collinearity angles. The data points depict the energy evolution of nearly out-of-plane (*squares*) and in-plane (*stars*) spin configurations, respectively (see Supplementary Figs. 3 and 4). The origin of the energy axis has been set with respect to the band-energy of the collinear (*θ* = 0) out-of-plane configuration. The *horizontal solid* and *dashed lines*, respectively, denote the minimum band-energy of the nearly out-of-plane and in-plane configurations, whose difference defines the effective anisotropy *K*_eff_. **d**, **e** *Schematic diagrams* of energy levels in zero magnetic field for the easy-plane case of the hcp hollow cluster (**d**, $K,\left( {\cal D} \right)  >0$) and the out-of-plane case of the fcc top, fcc hollow, and hcp top clusters (**e**, $K,\left( {\cal D} \right) < 0$), illustrating the effective spin model used for the simulation of the magnetization dynamics. Δ_01_ in **e** denotes the splitting between the ground and first excited states

Before we proceed with the detailed experimental investigation of these excitations, we describe spin-polarized density functional theory (DFT) calculations within the Korringa-Kohn-Rostoker Green function (KKR-GF) method (Fig. 2, see Methods, Supplementary Note 2 and Supplementary Fig. 2). Such calculations were performed in order to study the non-collinear GMC character of the trimers, which is expected from the large atomic mass and the Stoner enhancement of the substrate^22, 28^. We find, that, for all four cluster types, the magnetic moments of the three iron atoms are coupled approximately ferromagnetic with an exchange interaction *J* of several ten to hundred millielectron-volts (see Eq. 4 in Supplementary Note 2 for the used Hamiltonian and Supplementary Table 2 for the parameters). Moreover, there is a large cloud of more than a hundred spin-polarized Pt atoms in the vicinity of each cluster (Fig. 2a), adding up to a total magnetic moment of the whole cluster/substrate system of *m*_tot_ ~ 12 *μ*_B_ for fcc hollow/top and hcp top, and of *m*_tot_ ~ 13 *μ*_B_ for hcp hollow (see Supplementary Table 1 for the distribution of spin and orbital moments). Closer inspection shows that, while there are considerable non-collinearities of the spins in the polarization cloud, the Fe atom spins have tilting angles of only *θ* ≤ 4°, since the Dzyaloshinskii–Moriya contribution *D*_||_ to the exchange is an order of magnitude smaller than *J*. The only exception is the hcp top cluster, where $\frac{{{D_{||}}}}{J} \sim 0.5$ leads to tilting angles of up to *θ* ~ 17° (see Supplementary Table 2 and Supplementary Figs. 3 and 4). The clusters’ magnetizations have strong MAEs *K* on the order of one milli-electronvolt per Fe atom (Fig. 2b, c). Interestingly, the polarization cloud has an RKKY-like contribution to the MAE^26^, as revealed by its behavior as function of the number of Pt atoms considered in the collinear calculation (Fig. 2b). For a sufficient number of Pt atoms taken into account, the calculations predict a preferred orientation of the magnetization perpendicular to the surface (“out-of-plane”) for both fcc clusters, and a preferred orientation of the magnetization in the surface plane (“easy-plane”) for the hcp hollow cluster. Interestingly, the MAE favors the same orientation as in the system where the constituent atom is individually adsorbed on the surface^28^. The case of hcp top cluster is peculiar in that, although the MAE favors an easy-plane orientation by approximately 1.5 meV per adatom in the collinear calculation (see Fig. 2b), the strong non-collinearity emerging from the Pt substrate reverses the trend and favors instead a nearly out-of-plane orientation by  ~ 0.1 meV per adatom in the non-collinear calculation (Fig. 2c and Supplementary Fig. 5). The condition for the occurrence of this remarkable effect can be cast into the simple expression $\left| {\frac{{9D_{||}^2}}{{4J}}} \right|  >3K$ (see Eq. 21 in Supplementary Note 2), which shows that the effective anisotropy of the full trimer results from the competition between the non-collinear Dzyaloshinskii–Moriya contribution and the MAE. All in all, our DFT calculations reveal considerable MAEs of all four GMCs and a strong Dzyaloshinskii–Moriya–type coupling to the substrate conduction electrons, which is reflected by the non-collinear substrate polarization cloud.

### Effective spin model

In order to get a rough idea of the spin degrees of freedom of these GMCs, we map the DFT results onto an effective spin Hamiltonian, which will be later used to link the experiments to the ab-initio results. Considering the application of a magnetic field *B* in the out-of-plane direction, the lowest-order approximate effective spin Hamiltonian reads $\hat H = g{\mu _{\rm{B}}}{\hat S_z}B + {\cal D}\hat S_z^2$ ^11^. Here, ${\hat S_z}$ is the operator of the out-of-plane component of the total cluster effective spin, *g* is the Landé *g*-factor, *μ*_B_ is the Bohr magneton, and ${\cal D}$ is the longitudinal magnetic anisotropy constant. Since the Fe atoms and their nearest neighbor Pt atoms are strongly exchange-coupled among each other (see Supplementary Table 2), the GMC is treated in this Hamiltonian as a single object with a well-defined macro spin. Neglecting the slight non-collinearity of the three ferromagnetically coupled Fe atoms plus the neighboring Pt atoms in the cluster, the DFT calculated total magnetic moments result in a mapping to a total spin with quantum number of $S = - \frac{1}{2} + \sqrt {{{\left( {\frac{{{m_{{\rm{tot}}}}}}{{g{\mu _{\rm{B}}}}}} \right)}^2} + \frac{1}{4}} \approx 5$ or $\frac{{11}}{2}$ for the fcc hollow/top and hcp top GMCs, and of $S \approx 7$ or $\frac{{15}}{2}$ for the hcp hollow GMC. Here, we considered the *g*-factors extracted from ISTS as given in Table 1. Please note, that the effective spin model is a crude approximation for the case of magnetic impurities strongly interacting with a metallic substrate, since charge fluctuations can inhibit well-defined spin quantum numbers^33^. In the following, we choose the closest half-integer spin values ($\frac{{11}}{2}$ for fcc hollow/top and hcp top, and $\frac{{15}}{2}$ for hcp hollow) as we will see below that the hcp hollow cluster shows the Kondo effect, which strongly indicates a half integer spin ground state. However, the following discussion remains valid for the other choices of *S* by adjusting ${\cal D}$ accordingly. Taking into account the positive and negative values of ${\cal D}$ as estimated from the DFT-derived *K*-values for the easy-plane and out-of-plane clusters (see Table 1), the resulting qualitative energy level diagrams of the spin Hamiltonian for the two cases are shown in Fig. 2d, e, respectively, in zero magnetic field. We expect a degenerate ${S_z} = \pm \frac{1}{2}$ ground state for the hcp hollow cluster, and a degenerate ${S_z} = \pm \frac{{11}}{2}$ ground state for the fcc hollow/top and hcp top clusters. The former case is amenable to a quantum-mechanical superposition of the two $\frac{1}{2}$ spin states of the effective spin with the spin states of the substrate conduction electrons involving Kondo-correlations^33, 34^, while the latter typically favors magnetic bi-stability, similar to a classical magnet^5^. We, therefore, expect that the magnetic properties of the hcp hollow cluster will be drastically different from that of the other clusters.

**Table 1:** Determined values of the longitudinal anisotropy constant ${\cal D}$ and *g*-factors

GMC species	DFT	Spin excitations (ISTS)		Spin-dynamics (SPSTM)	
	${\cal D}$ (meV)	*g*	${\cal D}$	*g*	${\cal D}$
hcp hollow	+0.035	1.69 ± 0.22	—	—	—
hcp top	−0.01	2.18 ± 0.08	−0.05 ± 0.01	2.2	−0.025
fcc hollow	−0.25	1.86 ± 0.06	−0.07 ± 0.01	1.92	−0.05
fcc top	−0.03	2.12 ± 0.06	−0.09 ± 0.02	2.06	−0.05

The values of ${\cal D}$ expected from the DFT calculations were estimated from the calculated *K* (including non-collinear effects) via ${\cal D} = \frac{K}{{{S^2} - \frac{1}{4}}}$ assuming $S = \frac{{11}}{2}$ for hcp top, fcc hollow and top, and $S = \frac{{15}}{2}$ for hcp hollow, which are determined from the calculated magnetic moments together with the $g$-values used in the effective spin model. The values from the spin excitations are determined using the measured zero-field spin-excitation energies *Δ*_01_ via ${\cal D} = \frac{{{{\it{\Delta}} _{01}}}}{{2S - 1}}$, and the shifts of the excitations with *B* via $g = \frac{1}{{{\mu _{\rm B}}}}\frac{{{\rm{d}}{{\it{\Delta}} _{{\rm{01}}}}}}{{{\rm{d}}B}}$ (Supplementary Note 4, Supplementary Figs. 7, 8 and Supplementary Table 5). The ${\cal D}$ values determined from the spin-dynamics have been obtained by fitting the master equation model in conjuction with the effective spin Hamiltonian, using $S = \frac{{11}}{2}$ and the given *g*-factors, to the experimental data

### GMC spin excitations

**Fig. 3:**
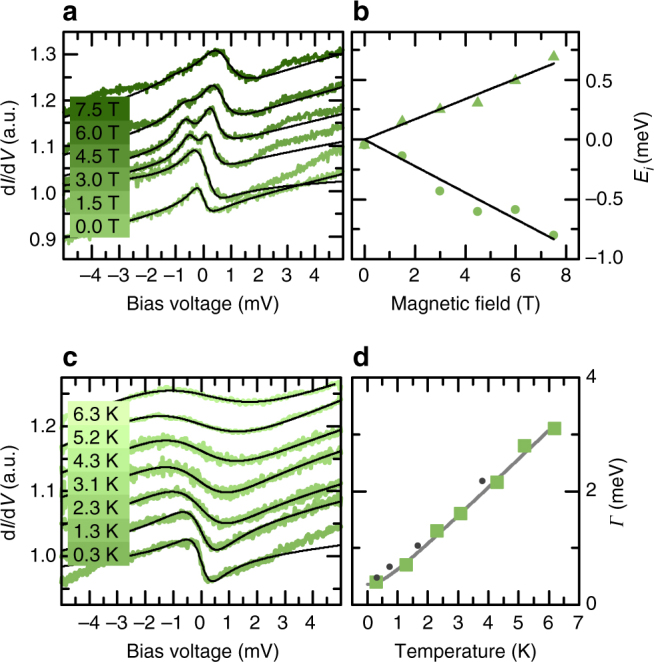
Kondo screening of the hcp hollow cluster. **a** *Colored lines* are spectra taken at the indicated magnetic fields on the hcp hollow cluster (*T* = 0.3 K). The *black lines* are fits to the sum of two Fano-functions (values of the full width at half maximum *Γ*≈0.5 meV and of the form factor *q*, see Supplementary Note 3, Supplementary Table 3, and Supplementary Fig. 6). **b** *Symbols* are the magnetic field dependent energetic positions of the Kondo resonances extracted from the fitted Fano-functions in **a**. The *solid lines* are linear fits through (*E*_*i*_,*B*) = (0,0) resulting in the *g*-factors of 1.46 and 1.91 for the positive and negative energy side, respectively. **c** Measured temperature dependence of the Kondo resonance (*colored lines*, *B* = 0T), together with fitted single Fano-functions (*black lines*, *E*_*i*_ ≈ 0 meV, values of *q*, see Supplementary Table 4). **d** Temperature dependency of the width *Γ* of the Kondo resonance extracted from the fitted Fano-functions in **c**, together with a fit to a power law (*gray line*) and to numerical renormalization group calculations for a spin-1/2 impurity in the strong coupling regime (*gray dots*, taken from ref. ^40^)

First, we experimentally investigated the excitations (Fig. 1c) of all four clusters by (I)STS as a function of magnetic field *B*, and for the hcp hollow GMC also as a function of temperature *T*. Indeed, for the hcp hollow cluster (Fig. 3a, c), we find a Fano-resonance right at the Fermi energy *E*_F_ (i.e., at *V* = 0 meV) that splits and broadens as a function of *B* and *T*, respectively. These are clear hallmarks for an emergent Kondo screening of the spin of this GMC^33–37^. The asymmetric lineshape of the Fano resonance is caused by the interference of a direct tunneling into the Kondo resonance, which arises as a consequence of the zero-energy spin flip scattering^38^, and other tunneling channels^39^. We can adequately fit the spectra to a sum of two Fano functions using the same form factor *q* and full width at half maximum *Γ*, but allowing for different energetic positions *E*_*i*_ of the Kondo resonances (lines in Fig. 3a, c, see the used Fano functions and parameters in Supplementary Note 3, Supplementary Tables 3 and 4, and Supplementary Fig. 6). The extracted values are used to quantify the field-induced splitting and temperature-driven broadening, as shown in Fig. 3b, d. Indeed, the resonance shifts linearly with *B* (Fig. 3b) with a *g*-factor close to 2 (*g*_hcp hollow_ ~ 1.7 ± 0.2), which we expect from the Kondo screening of the spin $\frac{1}{2}$ ground state of the easy-plane anisotropic hcp hollow cluster (Fig. 2d)^33, 34^. The dependence of *Γ* on temperature (Fig. 3d) nicely fits to a power law and to numerical renormalization group calculations for a spin-1/2 impurity^40^ (see Supplementary Note 3) using a Kondo temperature of *T*_K_ ~ 4.5 K. This value is one to two orders of magnitude smaller as compared to usual Kondo temperatures of single impurities on noble metal surfaces^36^, but has a similar size as that of atoms which are decoupled from the conduction electrons by thin insulators^34^ or that of Fe-hydrogen complexes on Pt(111)^33^. Our experimental findings thus prove Kondo correlations of the hcp hollow GMC with the substrate conduction electrons.

The magnetic-field evolution of the excitations of the fcc hollow/top and hcp top clusters behave drastically different (Fig. 1c, Supplementary Note 4, Supplementary Figs. 7 and 8). The spectra on these GMCs reveal a pair of features at energies symmetric to *E*_F,_ which linearly shift away from *E*_F_ with increasing magnetic field. This behavior is very reminiscent of systems with an out-of plane MAE^28^, which is excited from the ground state into the first excited state over the zero field energy splitting of ${{\it{\Delta}} _{01}} = {\cal D}\left( {2S - 1} \right)$ (Fig. 2e) that is linearly increasing by Zeeman energy. Note, however, that the shapes of the features deviate from simple steps, which probably indicates the limitation of the effective spin model (see Supplementary Note 4). By measuring the energetic positions of the excitations as a function of *B* (Supplementary Figs. 7 and 8 and Supplementary Table 5), we extract the corresponding longitudinal anisotropy constants ${\cal D}$ and *g*-factors close to 2, which are given in Table 1. The anisotropy constants for the two fcc and the hcp top clusters fit reasonably well with the constants estimated from the DFT calculations (see Table 1), substantiating the out-of-plane character of these GMCs.

### GMC spin dynamics

**Fig. 4:**
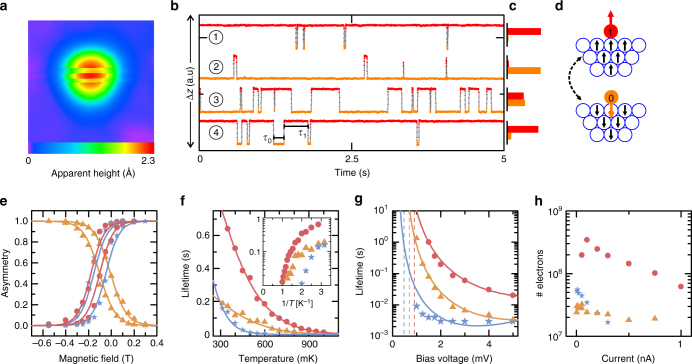
Magnetization switching of hcp top, fcc hollow, and fcc top clusters. **a** Spin-resolved constant-current image of a fcc top cluster taken with an STM tip sensitive to the out-of-plane component of the cluster magnetization, showing switching of the apparent height due to magnetization switching (3 × 3 nm^2^, *V* = 2 mV, *I* = 200 pA, *B* = 0 T, *T* = 0.3 K). **b** Spin-dependent telegraph signals measured at different magnetic fields and bias polarities on a fcc top cluster (*I* = 500 pA, *T* = 0.3 K, *B*_1_ = + 0.1 T, *V*_1_ = −5 mV, *B*_2_ = − 0.25 T, *V*_2_ = −5 mV, *B*_3_ = − 0.1 T, *V*_3_ = − 5 mV, *B*_4_ = − 0.1 T, *V*_4_ = + 5 mV). Two lifetimes *τ*_0_ and *τ*_1_ for the two magnetization states are indicated. **c** Voltage polarity- and magnetic field-dependent histograms (scale from 0 to 1) of the state-dependent lifetimes *τ*_0_ and *τ*_1_ illustrating favorability of the state 1 for positive field or positive bias (and state 0 for negative field or negative bias). **d** Sketch of the two magnetization states down (0) and up (1) comprised of the magnetization of the Fe cluster and a cloud of polarized Pt substrate atoms. **e** *Symbols* indicate measured magnetic field-dependent asymmetries ${\cal A}$ (*T* = 0.3 K, *I* = 500 pA) of hcp top (|*V*|=1.5 mV), fcc hollow (|*V*|=2 mV) and fcc top clusters (|*V*|=5 mV). **f** Measured temperature dependence of $\bar \tau$ at *B* = 0 T (hcp top: *V* = − 0.7 mV, *I* = 375 pA; fcc hollow: *V* = − 1.25 mV, *I* = 750 pA; fcc top: *V* = −2 mV, *I* = 750 pA). **g** Measured voltage dependence of $\bar \tau$ (*I* = 750 pA, *B* = 0 T, *T* = 0.3 K). *Solid lines* in **e**, **f**, and **g** are the corresponding model calculations using an effective spin of *S* = 11/2 with parameters of the magnetic anisotropy constants ${\cal D}$, the *g*-factors, and the tunnel couplings as given in Table 1 and the Methods section. *Dashed lines* in **g** indicate the energies Δ_01_ of the first excited state for each cluster type determined from ISTS (see Supplementary Table 5). **h** Average numbers of tunneling electrons ${\cal N} = \bar \tau \cdot I$ needed for a single switching event as a function of the current (*B* = 0 T, *T* = 0.3 K, *V* = 5 mV). The colors in **e**–**h** indicate the different cluster types hcp top (*blue*), hcp hollow (*orange*), and fcc top (*red*)

Due to the out-of-plane MAE of the fcc top/hollow and hcp top GMCs, we expect a bi-stable switching between the ${S_z} = \pm \frac{{11}}{2}$ ground states, which is observable if there are not too frequent tunneling electrons with an energy larger than Δ_01,_ which will drive the system across the barrier between these two states by sequential excitations (Fig. 2e)^5^. On the other hand, the emergent Kondo behavior of the hcp hollow GMC should lead to a quantum mechanical superposition of the ${S_z} = \pm \frac{1}{2}$ states of the effective spin with the spin states of substrate conduction electrons such that there is no observable switching of the magnetization. Indeed, using Cr coated tungsten tips, which are sensitive to the out-of-plane component of the GMC magnetization (Methods and Supplementary Fig. 9), we observe striped patterns in constant-current images taken on fcc top/hollow and hcp top clusters (shown exemplarily for the fcc top cluster in Fig. 4a), but not on the hcp hollow GMC. Such stripe patterns result from a random two-state telegraph noise^5, 31^, which is visible if the tip is positioned on-top of those clusters and the height is measured as a function of time (Fig. 4b). The telegraph signal can be unambiguously assigned to a statistical switching of the GMC between two spin states (0) and (1) of opposing out-of-plane magnetizations (Fig. 4d) by verifying that the asymmetry of the time-averaged occupational lifetimes ${\bar \tau _0}$ and ${\bar \tau _1}$ of the two states, defined by ${\cal A} = \frac{{{{\bar \tau }_1}}}{{{{\bar \tau }_0} + {{\bar \tau }_1}}}$, reverses by either reversing the out-of-plane oriented magnetic field *B*, or by reversing the out-of-plane spin-polarized tunneling current (bias polarity), which both drive the spin into one of the two states^5, 31^. We prove, that this is indeed the case for the fcc top/hollow and hcp top GMCs as shown in Fig. 4b, c. Similarly, full *B*-dependent loops of the asymmetry for two bias polarities recorded for all three clusters (Fig. 4e) show all the characteristics of a system randomly switching between two out-of-plane spin states and driven by the spin-pumping effect of the spin-polarized tunneling electrons^5^. Note, that there is a considerable shift of these asymmetry curves toward negative magnetic fields, which indicates an effective stray or exchange field of the tip^3, 41^ on the order of +0.1T. Moreover, the asymmetry curve recorded on the fcc hollow GMC is apparently inverted with respect to the vertical axis, which might be related with an inverted vacuum spin-polarization of this cluster as compared to the other two clusters, as sensed by the tip apex.

We consequently identify the two spin states (0) and (1) observed in the telegraph noise with the two ground states ${S_z} = + \frac{{11}}{2}$ and ${S_z} = - \frac{{11}}{2}$ of the fcc top/hollow and hcp top GMCs (Fig. 2e). In order to study the dynamics in these spin states in detail, we measure the telegraph noise as a function of bias voltage, tunneling current, and temperature (Fig. 4f–h). The temperature dependence of the average lifetime $\bar \tau = {\left( {\frac{1}{{{{\bar \tau }_0}}} + \frac{1}{{{{\bar \tau }_1}}}} \right)^{ \!\! - 1}}$ (Fig. 4f) is slightly different for all three clusters and on the order of a few tens of seconds at *T* = 300 mK. It reveals an exponential decay with increasing temperature, which can be assigned to a quasi-classical Arrhenius-like behavior that has been observed for larger scale Fe islands^31^ and non-*C*_*3v*_ *-*symmetric clusters at larger temperatures^5^. The absence of any shoulder in the temperature dependence down to our lowest measurement temperature indicates, that transversal anisotropies, which could induce quantum tunneling processes, are negligible here^5^. Furthermore, as shown in Fig. 4g, $\bar \tau$ increases over more than two decades if the bias voltage approaches the excitation thresholds $\frac{{{{\it{\Delta}} _{01}}}}{e}$ determined from ISTS (Supplementary Table 5), and finally gets too long for a statistically relevant measurement. This behavior is indeed expected, considering that, without quantum tunneling through the barrier, the spin state of the GMC can only overcome the barrier by sequential excitation processes, which need either tunneling or substrate electrons with energies larger than Δ_01_. Indeed, we can consistenly reproduce all measurements by using a master equation approach (see Methods) in conjuction with the effective spin Hamiltonian given above, with $S = \frac{{11}}{2}$, the *g*-factors extracted from ISTS (see Table 1) and fitting the longitudinal magnetic anisotropy parameter ${\cal D}$ (lines in Fig. 4e–g). The resulting magnetic anisotropies given in Table 1 agree with the parameters determined from ISTS remarkably well, giving a final support for the quasi-classical spin behavior of the fcc top/hollow and hcp top GMCs, in stark contrast to the Kondo character of the hcp hollow GMC.

### GMCs as two-bit registers and four-state memories

**Fig. 5:**
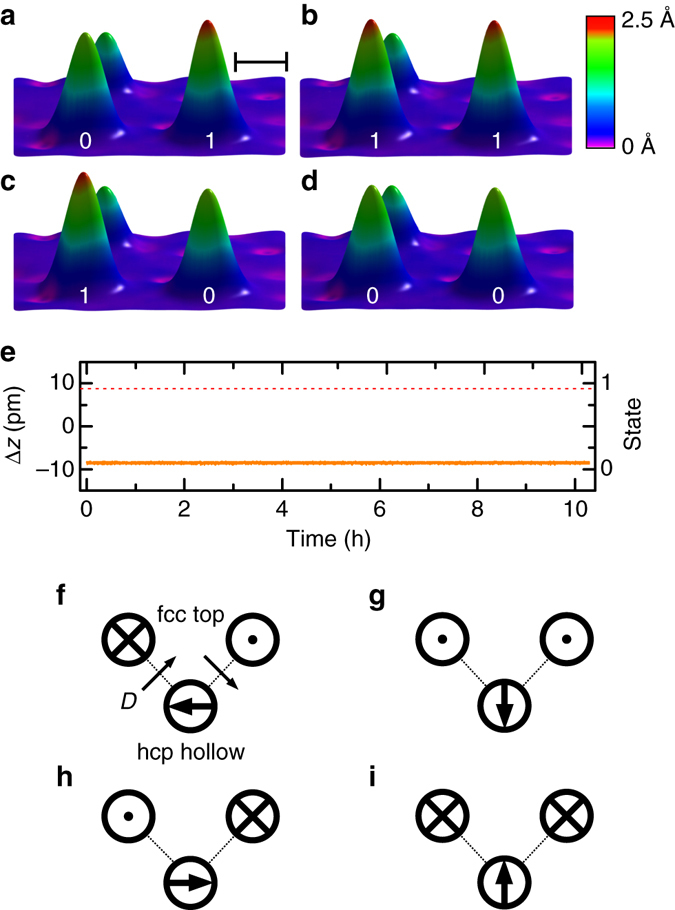
Two-bit register and four-state memory based on giant moment clusters. **a**–**d** Spin-resolved constant-current images of two fcc top clusters in the four possible spin states (01), (11), (10), and (00), where 0 and 1 correspond to downwards and upwards pointing magnetization, respectively (imaging parameters: *V* = −1 mV, *I* = 500 pA, *B* = 0 T, *T* = 0.3 K). The Fe atom in the back serves as a marker for the apparent height. The *scale bar* defines a length of 1 nm. Between the images, the tip was positioned on top of the cluster whose state was intended to be changed, the bias was slowly increased until the state switched, and then quickly decreased to the imaging parameter. **e** The long-term stability of one of the two magnetic bits is shown by measuring the height of the cluster (*orange trace*) in the state 0 over more than 10 h (*V* = −0.7 mV, *I* = −50 pA, *B* = 0 T, *T* = 0.3 K), without a single switch into the state 1, whose height reference is given by the *red dotted line*, which was determined from the magnetic contrast in images **a**–**d**. **f**–**i**, *Top view* schematic diagram of a possible memory with four different realized spin states on a hcp hollow cluster. The two *upper circles* in each panel illustrate two fcc top clusters prepared in magnetization states *up* (*dot*) or *down* (*cross*). The lower circle in each panel signifies a hcp hollow cluster (or a hcp atom), which is forced into one of the four spin states indicated by the *thick arrow*, as dictated by the Dzyaloshinskii–Moriya interaction *D* (orientation indicated by the two *thin arrows*) to the two neighboring clusters

As suggested by Fig. 4g, a spin-polarized tunneling current of sufficiently small bias below the excitation threshold $\frac{{{{\it{\Delta}} _{01}}}}{e}$ could be used to “read” the quasi-classical spin state of the fcc top/hollow and hcp top GMCs with negligible disturbance. On the other hand, a current biased above this threshold could be used to switch the spin state and thereby “write” information. This is verified by building a two-bit register from two of the magnetically most stable fcc top GMCs located next to each other in a distance of only 2.5 nm (Fig. 5a–d). Indeed, we were able to write the state (0) or (1) into the desired GMC by application of a spin-polarized tunneling current driven by a voltage-ramp crossing $\frac{{{{\it{\Delta}} _{01}}}}{e}$ that was switched off when the GMC switched from (0) to (1), or vice versa, at wish, without changing the state of the neighboring GMC. The consecutive read out of the four possible states (01), (11), (10), and (00) of the two-bit register written like this is shown in Fig. 5a–d. Furthermore, we demonstrated, that such prepared states are stable in time by reading the spin state of one of the CMCs prepared in state (0), while the other was in state (1), over time. Figure 5e shows that the state is stable for at least 10 h, after which we had to stop the measurement due to restrictions imposed by the experimental facility.

Since the GMCs are strongly coupled to a heavy metallic substrate that was experimentally shown to mediate Dzyaloshinskii–Moriya interactions tunable by the distance^22^, the realization of a two-bit register of spins, demonstrated here, offers further perspectives for spin-based information processing schemes. One possible scheme made from the two-bit register in Fig. 5a–d and an additional hcp-hollow cluster (or alternatively an Fe atom on hcp adsorption site which has easy-plane anisotropy^33^) in close to perpendicular geometry is shown in Fig. 5f. The hcp-hollow cluster should be assembled at a distance where the substrate mediated interaction is strong enough to quench the Kondo screening, permitted by its small Kondo temperature (*k*_B_ *T*_K_ ~ 0.4 meV). Since the Dzyaloshinskii–Moriya vector **D** has a strong contribution in the plane and perpendicular to the dotted line (see Fig. 5f)^22^, the spin of the hcp hollow cluster (or hcp atom) can be forced into the four different orientations shown in Fig. 5f–i depending on the four possible states of the two-bit register.

We finally compare the magnetic stability of the three-atomic fcc top GMC investigated here with the system of five Fe atoms constructed on the (111) surface of copper^5^. As shown in Fig. 4h, under writing conditions, a number of about ${\cal N} = \bar \tau \cdot I = {10^8}$ tunneling electrons (at 0.75 nA current and 5 mV bias, slightly depending on the current) is needed for a single switching event of the fcc top GMC. We investigated other fcc top clusters using different tips and found that ${\cal N}$ can be as large as 10^9^ (0.75 nA, 10 mV), where the variation between clusters results from small variations in the MAE due to electronic inhomogeneity of the substrate^5^. This number is about five to ten times smaller than that needed to switch Fe_5_ on Cu(111) at comparable tunneling parameters. The increased sensitivity under writing conditions might be explained by the smaller number of atoms in the GMC investigated here, leading to a smaller spin and consequently to a smaller number of states separating the two spin states (0) and (1). Fully understanding why Fe_3_ on Pt(111) still is a magnetically stable GMC requires a detailed analysis of diverse effects, such as symmetry protection^14^ or quantum spin-fluctuations. For instance, the higher symmetry of the Fe_3_ on Pt(111) GMC leeds to negligible transversal anisotropies efficiently reducing quantum mechanical tunneling of the spin through the MAE barrier, which is not the case for the less symmetric Fe_5_ on Cu(111) system. Moreover, quantum spin-fluctuations can be suppressed if the hybridization with the substrate is less effective^42^, which appears to be the case for Pt(111) in comparison to Cu(111), according to our preliminary results. These effects are likely to be the reason for the fact, that the Fe_3_ on Pt(111) GMC can still serve as a stable magnetic bit under reading conditions, while it needs less electrons to switch its state under writing conditions.

## Discussion

In summary, we experimentally demonstrated that a GMC of only three Fe atoms strongly coupled to a heavy-element substrate constitutes a stable quasi-classical magnet suitable for storing information for hours. Due to the strong coupling of the magnetic bit to a substrate that features non-collinear RKKY-interactions^22^, the spin-information can be processed with large variability, as we demonstrate by the scheme of a four-state memory. Moreover, the chosen substrate platinum is a prototypical strong spin-orbit coupling material widely used in the research on spintronics technology concepts. Therefore, the few-atom magnet we have realized here is an ideal model system to study the down-scaling of such concepts, as, for example, the application of spin-orbit torques for writing information^43, 44^, to the limit of single atoms.

## Methods

### Experimental procedures

All measurements have been performed under ultra-high vacuum conditions in a home-built low-temperature scanning tunneling microscope facility were the magnetic field *B* is applied perpendicular to the sample surface^45^. The Pt(111) single crystal was cleaned in situ by argon ion sputtering and annealing cycles and a final flash as described in ref. ^28^. While the sample cooled down to room temperature after the flash, a fraction of a monolayer Co was deposited from an e-beam heated rod resulting in the decoration of the Pt step edges and terraces with Co stripes and islands, respectively, of one atomic layer height^3, 27^ (see Supplementary Fig. 9). After the sample was cooled to *T* ~ 4 K,  ~ 1% of a monolayer Fe was co-deposited from an e-beam heated rod. During deposition, the sample temperature did not exceed *T* ~ 10 K resulting in a statistical distribution of single Fe atoms on the Pt terraces (Fig. 1a and Supplementary Fig. 9).

Constant-current images were recorded at a tunnelling current *I* with a bias voltage *V* applied to the sample. The Fe clusters have been formed by lateral manipulation of Fe atoms using a current of *I* = 25–35 nA with voltages of *V* = 0.9–1.3 mV^2, 7^. For spin-resolved scanning tunneling microscopy (SPSTM), we coated flashed tungsten tips with about 50 monolayers of Cr^32^. Spin contrast with a sensitivity to the out-of-plane component of the sample magnetization was then achieved by picking up Fe atoms until a strong magnetic contrast was observed on the remanently out-of-plane magnetized Co stripes or islands^3, 27^ (Supplementary Fig. 9). For the investigation of the spin excitations by spin-averaged ISTS we dipped the tip into the Pt substrate until the telegraph signal on the out-of-plane clusters vanished and the spectrum taken on the Pt was feature-less within the voltage range used for ISTS. Then, ISTS was performed by positioning the tip on top of the GMC, adding a modulation voltage *V*_mod_ = 80 μV (r.m.s.) of frequency *f*_mod_ = 4142 Hz to *V*, stabilizing the tip at *I*_stab_ = 2 nA and *V*_stab_ = 5 mV, switching the feedback off, ramping the bias voltage and recording the $\frac{{{\rm{d}}I}}{{{\rm{d}}V}}$ signal using a lock-in amplifier. For the investigation of the spin dynamics of the GMCs by SPSTM, we moved an out-of-plane spin-sensitive tip on top of the cluster, waited until the scanner creep was negligible, and recorded the tip height *z* as a function of time in constant-current mode^5^.

### Ab initio DFT calculations

For the ab initio DFT calculations, we have used the KKR-GF method including spin-orbit coupling within the local-spin-density approximation^46, 47^. The Pt(111) hollow and top clusters have been modeled by a slab containing 24 and 64 Pt atoms, respectively, in both cases augmented by two vacuum regions. The relaxed distances of the Fe trimers toward the surface have been calculated by means of the QUANTUM-ESPRESSO package^48^. The MAE and the non-collinear energy landscape of the four types of trimers have been evaluated by band energy differences following the magnetic force theorem^49^, while the magnetic interactions among the Fe atoms have been computed using the generalized Liechtenstein formula^50–52^ and a fine mesh of 200 × 200 × 1 *k* points. For further details, we refer the reader to the Supplementary Note 2.

### Master equation model

In order to simulate the GMC dynamics, we used the master equation model in conjunction with the effective spin Hamiltonian as described in ref. ^5^. Additionally, we took into account a bias voltage dependent change in the ratio between the coupling of the cluster to the tip, *v*_tip_, and to the surface, *v*_surface_, which we observed for the clusters investigated here. To this end, we assumed an exponential dependence on the bias voltage *V* via ${\left( {\frac{{{v_{{\rm{tip}}}}}}{{{v_{{\rm{surface}}}}}}} \right)_V} = {\left( {\frac{{{v_{{\rm{tip}}}}}}{{{v_{{\rm{surface}}}}}}} \right)_{V_T}} \times {{\rm e}^{ - \kappa \cdot \left( {V - {V_T}} \right)}}$, where ${\left( {\frac{{{v_{{\rm{tip}}}}}}{{{v_{{\rm{surface}}}}}}} \right)_{{V_T}}}$ is the ratio determined from the fit of the temperature dependence of the lifetime measured at voltage *V*_*T*_ (Fig. 4f). The tip retraction parameter *κ* then follows from the fitting of the voltage dependence of the lifetime (Fig. 4g), resulting in ${\left( {\frac{{{v_{{\rm{tip}}}}}}{{{v_{{\rm{surface}}}}}}} \right)_{{V_T}}} = 0.085$ and *κ* = 0.15 for hcp top, ${\left( {\frac{{{v_{{\rm{tip}}}}}}{{{v_{{\rm{surface}}}}}}} \right)_{{V_T}}} \!\! = 0.095$ and *κ* = 0.1 for fcc hollow, and ${\left( {\frac{{{v_{{\rm{tip}}}}}}{{{v_{{\rm{surface}}}}}}} \right)_{{V_T}}} \!\!= 0.07$ and *κ* = 0.08 for the fcc top cluster. Since $\frac{{{v_{{\rm{tip}}}}}}{{{v_{{\rm{surface}}}}}}$ additionally depends on the current, the fitted voltage dependence slightly deviates from the experimental data for the hcp top cluster. $\frac{{{v_{{\rm{tip}}}}}}{{{v_{{\rm{surface}}}}}}$ varies between 0.01 and 0.022 for the field dependent asymmetry fits (Fig. 4e).

### Data availability

The authors declare that the main data supporting the findings of this study are available within the article and its Supplementary Information files. Extra data are available from the corresponding author upon reasonable request.

## Electronic supplementary material

Supplementary Information

## References

[1] Chappert C, Fert A, Van Dau FN (2007). The emergence of spin electronics in data storage. Nat. Mater..

[2] Eigler DM, Schweizer EK (1990). Positioning single atoms with a scanning tunnelling microscope. Nature.

[3] Meier F, Zhou L, Wiebe J, Wiesendanger R (2008). Revealing magnetic interactions from single-atom magnetization curves. Science.

[4] Heinrich AJ, Gupta JA, Lutz CP, Eigler DM (2004). Single-atom spin-flip spectroscopy. Science.

[5] Khajetoorians AA (2013). Current-driven spin dynamics of artificially constructed quantum magnets. Science.

[6] Loth S, Baumann S, Lutz CP, Eigler DM, Heinrich AJ (2012). Bistability in atomic-scale antiferromagnets. Science.

[7] Khajetoorians AA, Wiebe J, Chilian B, Wiesendanger R (2011). Realizing all-spin–based logic operations atom by atom. Science.

[8] Baumann S (2015). Electron paramagnetic resonance of individual atoms on a surface. Science.

[9] Delgado F, Fernández-Rossier J (2017). Spin decoherence of magnetic atoms on surfaces. Prog. Surf. Sci..

[10] Gambardella P (2003). Giant magnetic anisotropy of single cobalt atoms and nanoparticles. Science.

[11] Gatteschi, D. & Sessoli, R. *Molecular Nanomagnets* (Oxford Univ. Press, 2006)

[12] Kondo J (1964). Resistance minimum in dilute magnetic alloys. Prog. Theor. Phy..

[13] Donati F (2016). Magnetic remanence in single atoms. Science.

[14] Hübner C, Baxevanis B, Khajetoorians AA, Pfannkuche D (2014). Symmetry effects on the spin switching of adatoms. Phys. Rev. B.

[15] Singha A (2016). Magnetic hysteresis in Er trimers on Cu(111). Nano Lett..

[16] Balashov T (2014). Dynamic magnetic excitations in 3d and 4f atoms and clusters. Surf. Sci..

[17] Rau IG (2014). Reaching the magnetic anisotropy limit of a 3d metal atom. Science.

[18] Paul W (2010). Control of the millisecond spin lifetime of an electrically probed atom. Nat. Phys.

[19] Heinrich BW, Braun L, Pascual JI, Franke KJ (2013). Protection of excited spin states by a superconducting energy gap. Nat. Phys..

[20] Khajetoorians AA (2010). Detecting excitation and magnetization of individual dopants in a semiconductor. Nature.

[21] Zhou L (2010). Strength and directionality of surface Ruderman-Kittel-Kasuya-Yosida interaction mapped on the atomic scale. Nat. Phys..

[22] Khajetoorians AA (2016). Tailoring the chiral magnetic interaction between two individual atoms. Nat. Commun..

[23] Błoński P (2010). Magnetocrystalline anisotropy energy of Co and Fe adatoms on the (111) surfaces of Pd and Rh. Phys. Rev. B.

[24] Oswald A, Zeller R, Dederichs PH (1986). Giant moments in palladium. Phys. Rev. Lett..

[25] Šipr O, Bornemann S, Minár J, Ebert H (2010). Magnetic anisotropy of Fe and Co adatoms and monolayers: need for a proper treatment of the substrate. Phys. Rev. B.

[26] Bouhassoune M, Dias MdS, Zimmermann B, Dederichs PH, Lounis S (2016). RKKY-like contributions to the magnetic anisotropy energy: 3d adatoms on Pt(111) surface. Phys. Rev. B.

[27] Meier F (2011). Spin polarization of platinum (111) induced by the proximity to cobalt nanostripes. Phys. Rev. B.

[28] Khajetoorians AA (2013). Spin excitations of individual Fe atoms on Pt(111): Impact of the site-dependent giant substrate polarization. Phys. Rev. Lett..

[29] Mydosh, J. A. *Spin Glasses: An Experimental Introduction* (Taylor & Francis Ltd, 1993)

[30] Jamneala T, Madhavan V, Crommie MF (2001). Kondo response of a single antiferromagnetic chromium trimer. Phys. Rev. Lett..

[31] Krause S, Berbil-Bautista L, Herzog G, Bode M, Wiesendanger R (2007). Current-induced magnetization switching with a spin-polarized scanning tunneling microscope. Science.

[32] Khajetoorians AA (2011). Itinerant nature of atom-magnetization excitation by tunneling electrons. Phys. Rev. Lett..

[33] Khajetoorians AA (2015). Tuning emergent magnetism in a Hund’s impurity. Nat. Nanotechnol..

[34] Otte AF (2008). The role of magnetic anisotropy in the Kondo effect. Nat. Phys..

[35] Madhavan V, Chen W, Jamneala T, Crommie MF, Wingreen NS (1998). Tunneling into a single magnetic atom: Spectroscopic evidence of the Kondo resonance. Science.

[36] Nagaoka K, Jamneala T, Grobis M, Crommie MF (2002). Temperature dependence of a single Kondo impurity. Phys. Rev. Lett..

[37] Zhang Y-h (2013). Temperature and magnetic field dependence of a Kondo system in the weak coupling regime. Nat. Commun..

[38] Suhl H (1965). Dispersion theory of the Kondo effect. Phys. Rev..

[39] Újsághy O, Kroha J, Szunyogh L, Zawadowski A (2000). Theory of the Fano resonance in the STM tunneling density of states due to a single Kondo impurity. Phys. Rev. Lett..

[40] Costi TA (2000). Kondo effect in a magnetic field and the magnetoresistivity of Kondo alloys. Phys. Rev. Lett..

[41] Yan S, Choi D-J, Burgess JAJ, Rolf-Pissarczyk S, Loth S (2015). Control of quantum magnets by atomic exchange bias. Nat. Nanotechnol..

[42] Ibañez-Azpiroz J, dos Santos Dias M, Blügel S, Lounis S (2016). Zero-point spin-fluctuations of single adatoms. Nano Lett..

[43] Brataas A, Hals KMD (2014). Spin-orbit torques in action. Nat. Nanotechnol..

[44] Miron IM (2011). Perpendicular switching of a single ferromagnetic layer induced by in-plane current injection. Nature.

[45] Wiebe J (2004). A 300 mk ultra-high vacuum scanning tunneling microscope for spin-resolved spectroscopy at high energy resolution. Rev. Sci. Instrum..

[46] Papanikolaou N, Zeller R, Dederichs PH (2002). Conceptual improvements of the KKR method. J. Phys. Condens. Matter.

[47] dos Santos Dias M, Schweflinghaus B, Blügel S, Lounis S (2015). Relativistic dynamical spin excitations of magnetic adatoms. Phys. Rev. B.

[48] Paolo G (2009). Quantum espresso: A modular and open-source software project for quantum simulations of materials. J. Phys. Condens. Matter.

[49] Weinert M, Watson RE, Davenport JW (1985). Total-energy differences and eigenvalue sums. Phys. Rev. B.

[50] Liechtenstein AI, Katsnelson MI, Gubanov VA (1984). Exchange interactions and spin-wave stiffness in ferromagnetic metals. J. Phys. F: Metal Phys..

[51] Polesya S (2010). Finite-temperature magnetism of Fe_x_Pd_1-x_ and Co_x_Pt_1-x_ alloys. Phys. Rev. B.

[52] Udvardi L, Szunyogh L, Palotás K, Weinberger P (2003). First-principles relativistic study of spin waves in thin magnetic films. Phys. Rev. B.

