# Supplementary material for: A gateway towards non-collinear spin processing using three-atom magnets with strong substrate coupling
Source: Nat Commun. 2017 Sep 21;8:642. doi: 10.1038/s41467-017-00506-7 (PMC5608713; doi:10.1038/s41467-017-00506-7)
Supplement: Supplementary file 1 — Supplementary Information [file 41467_2017_506_MOESM1_ESM.pdf]

File name: Supplementary Information
Description: Supplementary Notes, Supplementary Figures, Supplementary Tables and Supplementary References

Supplementary Note 1 | Determination of the giant moment cluster structure

In order to determine the internal structure of the GMCs, we assembled several individual fcc and hcp atoms close to the built GMCs, as shown in the top of Supplementary Fig.1 exemplarily for one of the GMCs. The stacking type (fcc, hcp) of the individual Fe atoms is unambiguously identified by their characteristic ISTS signature [1]. Thereby, we can put a lattice of Pt surface atoms with assigned fcc and hcp adsorption sites on top of the GMCs topography (Supplementary Fig.1), and conclude that it contains either three fcc or three hcp atoms on nearest neighboring adsorption sites. Moreover, the GMC is not exactly round shaped, but slightly has the form of a downwards pointing triangle. We therefore conclude that this GMC is an fcc top cluster. The same procedure leads to the identification of the internal structure of the three other GMCs, which are shown schematically in the bottom of Supplementary Fig.1.

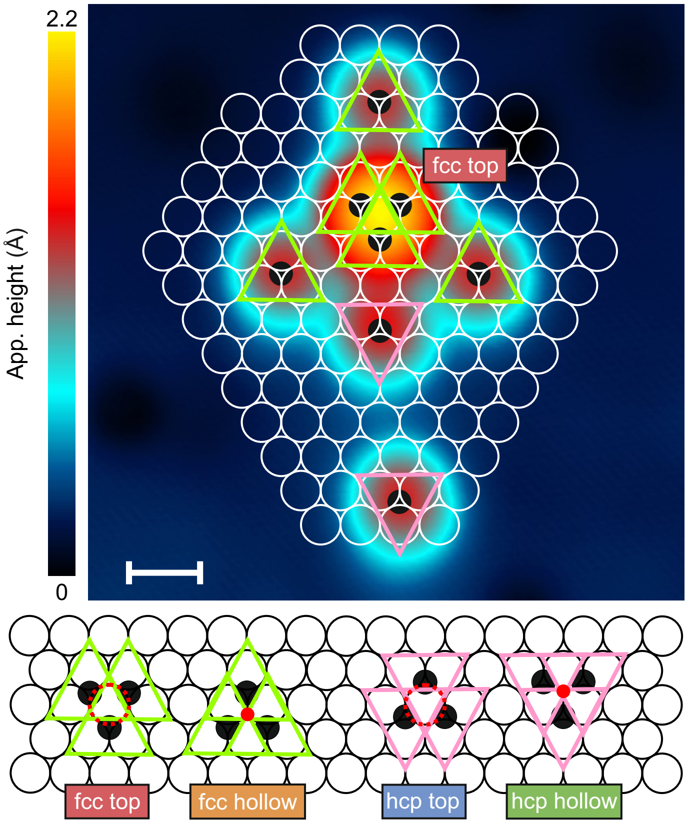

Supplementary Figure 1 | Giant moment cluster structure. Constant-current image ($4 \times 4\,\mathrm{nm}^2$) of an assembly of a fcc top GMC, three individual fcc atoms (marked with green triangles), and two individual hcp atoms (marked with pink triangles) close by. The scale bar defines a length of $0.5\,\mathrm{nm}$. Tunneling parameters: $V = 5\,\mathrm{mV}$, $I = 500\,\mathrm{pA}$, $B = 0\,\mathrm{T}$, $T = 0.3\,\mathrm{K}$.

Supplementary Note 2 | Density Functional Theory

Computational details Density functional theory (DFT) calculations were performed in the framework of the Korringa-Kohn-Rostoker Green function (KKR-GF) real-space approach in the atomic sphere approximation with full charge density and including spin-orbit coupling [2,3]. The exchange and correlation effects were taken into account using the local spin-density approximation (LSDA) as parametrized by Vosko, Wilk and Nusair [4]. The Pt(111) surface was modeled by a slab of 24 Pt layers augmented by two vacuum regions 21.1 Å thick each, using the experimental lattice constant (a = 3.92 Å). To capture all the details of the hcp top and fcc top structures we have performed additional calculations using a slab containing 64 Pt atoms, corresponding to a 3x3 two-dimensional unit cell and 6 Pt layers (see next subsection and Supplementary Figure 2 for more details). The Fe trimers were constructed by cutting out a real space cluster centered on the positions to be occupied by the Fe atoms, which is embedded into the Pt substrate (see Fig. 2a of the main text). We have made sure that our results are converged with respect to the number of Pt atoms in the cluster, given that the MAE is found to be very sensitive to the Pt spin-polarization. Following this approach, we have modeled the four different structures analyzed in the main text, namely hcp hollow (194 Pt atoms in the cluster), hcp top (188 Pt atoms), fcc hollow (156 Pt atoms) and fcc top (170 Pt atoms).

For each of the four configurations, the vertical relaxation of the trimer was calculated using the QUANTUM-ESPRESSO package [5], imposing a relaxation criterion whereby the vertical force exerted on individual Fe atoms and Pt atoms of the surface layer is $<10^{-4}$ Ry a.u.$^{-1}$. The computational modeling of the system was done employing the repeated slab approach considering 86 atoms per unit cell, an energy cutoff of 40 Ry, a Gamma-point reciprocal-space mesh and ultrasoft fully relativistic pseudopotentials. In the hcp hollow and fcc hollow configurations, the trimer relaxes vertically 17.5% towards the surface (0% corresponds to the ideal interlayer separation in bulk, $a/\sqrt{3} = 2.26$ Å), and the height of the Pt surface layer remains approximately constant. In contrast, in the hcp top and fcc top configurations, one of the surface Pt atoms is shifted with respect to the rest of the Pt surface layer, as schematically illustrated in Supplementary Figure 2. Note that this particular Pt atom lies underneath the center of the Fe trimer and is therefore the only substrate atom that is a nearest neighbor of every Fe adatom. Finally, total energy calculations predict that the fcc and hcp hollow configurations are the energetically most stable ones (the calculated energy difference between the two is below our numerical precision), followed by fcc top (35 meV/adatom higher) and hcp top (45 meV/adatom higher).

Magnetic moments, orbital moments and exchange parameters The magnetic moments obtained using the KKR-GF approach are summarized in Supplementary Table 1. In all cases, we find that the magnetic moments of individual Fe adatoms are close to 3.5 μ_B, while the surrounding Pt atoms have a substantial total contribution that ranges between 1.4 and 2.2 μ_B depending on the cluster type. It is particularly noteworthy that in the hcp top and fcc top configurations, the Pt atom with 10% vertical distance height (see previous subsection and Supplementary Figure 2) becomes strongly spin-polarized by 0.23 μ_B, a factor 3 larger than its closest Pt neighbors. Finally, our calculations show a substantial orbital magnetic moment of both Fe adatoms and the Pt cluster.

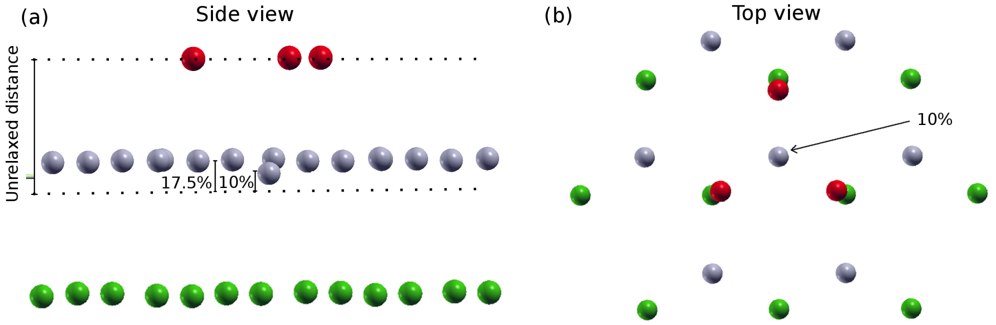

Supplementary Figure 2 | Structure optimization. (a) and (b) Respectively, side and top view of the relaxed structure of the hcp top cluster (only the trimer plus the first two Pt layers are shown for clarity). Fe adatoms, Pt surface atoms and Pt atoms from the second layer are symbolized by red, grey and green spheres, respectively. The surface Pt atom underneath the center of the Fe trimer relaxes to a different height as compared to the rest of the surface layer.

The calculated magnetic exchange interaction parameters of the Fe trimer are summarized in Supplementary Table 2. For completeness, we have included calculations performed in the hcp top and fcc top structures where the single Pt surface atom underneath the center of the trimer was not shifted with respect to the rest of surface Pt atoms; we add a label 'c. h.' (constant height) to denote this structures. The MAE, denoted by K, has been calculated *ab initio* following the magnetic force theorem [6]. The rest of the parameters have been evaluated based on the generalized Lichtenstein formula [7–9]. Due to the C_{3v} symmetry of the system the three Dzyaloshinskii–Moriya (DM) vectors are fully specified by two parameters, $D_\parallel$ and $D_\perp$ (see Suppl. Figs. 3 and 4):

$$\mathbf{D}_{12} = (0, -D_\parallel, D_\perp), \tag{1}$$

$$\mathbf{D}_{23} = (-\frac{\sqrt{3}}{2}D_\parallel, \frac{1}{2}D_\parallel, D_\perp), \tag{2}$$

$$\mathbf{D}_{31} = (\frac{\sqrt{3}}{2}D_\parallel, \frac{1}{2}D_\parallel, D_\perp). \tag{3}$$

In addition, we have employed a scheme [8] that allows to incorporate the effect of the spin-polarized Pt substrate into the direct exchange coupling J, yielding a renormalized parameter J_{renorm}. We

	$m_{\text{sp., Fe}}$ (μ_B)	$m_{\text{orb., Fe}}$ (μ_B)	$m_{\text{sp., Pt cls.}}$ (μ_B)	$m_{\text{orb., Pt cls.}}$ (μ_B)
hcp hollow	3.46	0.12	2.20	0.22
hcp top	3.33	0.12	2.03	0.08
fcc hollow	3.29	0.14	1.39	0.13
fcc top	3.28	0.12	1.72	0.12

Supplementary Table 1 | Magnetic moments. Calculated magnetic moments for the four different configurations considered in the main text. $m_{\text{sp., Fe}}$ and $m_{\text{orb., Fe}}$ denote respectively the magnetic and orbital moment per Fe adatom, while $m_{\text{sp., Pt cls.}}$ and $m_{\text{orb., Pt cls.}}$ refer to the magnetic and orbital moment of the full Pt cluster, respectively.

find that all the calculated J's shown in Supplementary Table 2 are fairly large and negative, thus favouring a ferromagnetic coupling between the spins of the Fe trimer. Moreover, the renormalization induced by the Pt substrate has the net effect of increasing the magnitude of the J's by approximately 20-30%, thus favouring even more the ferromagnetic coupling. In comparison, the DM interaction is nearly one order of magnitude smaller, except for the case of the hcp top structure, both in the normal and c. h. case. It is noteworthy that the $J/D_{\|}$ ratio varies by nearly 30%, thus revealing a large effect induced by the single shifted Pt atom.

Non-collinear spin structure Our DFT calculations have found a close competition between two almost ferromagnetic, slightly non-collinear spin configurations for the four trimers. In one of them, the magnetic moments point mainly along the out-of-plane direction with a non-collinearity polar angle θ as illustrated in Supplementary Figure 3. In the other one, shown in Supplementary Figure 4, the magnetic moments point nearly in-plane with two of them opening with a non-collinearity angle denoted as x. The corresponding band energy calculations, denoted by ΔE_b, have been performed in two steps. Firstly, we have converged the solution to a strictly ferromagnetic out-of-plane configuration. Secondly, we have performed band energy calculations employing the magnetic force theorem [6] of canted configurations with varying opening angle, both for the setup illustrated in Supplementary Figures 3 and 4. The results are illustrated in Supplementary Figure 5 for all the configurations, including the 'c. h.' discussed in Supplementary Table 2. This figure shows that the nearly out-of-plane configuration is the most stable one for hcp top, fcc hollow and fcc top structures. In the particular case of hcp top, we note that it is necessary to consider the structure with the single shifted Pt atom in order to obtain the correct orientation of the magnetic moments that is in accordance with experimental measurements. Furthermore, we emphasize the importance of the energy minimization induced by the non-collinearity for the hcp top cluster; even though the ferromagnetic alignment favors the in-plane direction by nearly 1.5 meV/adatom, the energy gained by the non-collinearity reverses the trend and favours instead a nearly out-of-plane orientation with large canted angles of approximately $\theta = 17°$ by ~ 0.1 meV/adatom. In comparison, the fcc top and fcc hollow configurations show a small non-collinearity angle $\theta < 4°$. On the opposite side, the favoured configuration for the hcp hollow cluster type is the one with

	K (meV/ad.)	J (meV)	$J_{\text{renorm.}}$ (meV)	$D_{\|}$ (meV)	$D_{\perp}$ (meV)	θ, x (°)
hcp hollow	0.65	-52	-71	8.7	-5.0	4.1
hcp top	1.51	-34	-51	21.1	-3.4	17.2
hcp top c. h.	1.23	-34	-47	10.7	-4.4	6.5
fcc hollow	-2.31	-96	-116	0.5	-3.6	~ 0
fcc top	-0.12	-71	-91	4.5	-0.7	3.9
fcc top c. h.	-0.21	-82	-108	2.5	-1.3	1.5

Supplementary Table 2 | Calculated exchange parameters. The label 'c. h.' denotes the top configurations where the Pt surface atom underneath the center of the trimer was not shifted with respect to the rest of surface Pt atoms. The non-collinearity angle θ, x refers to the angle of the most stable non-collinear configuration, see Supplementary Figure 5.

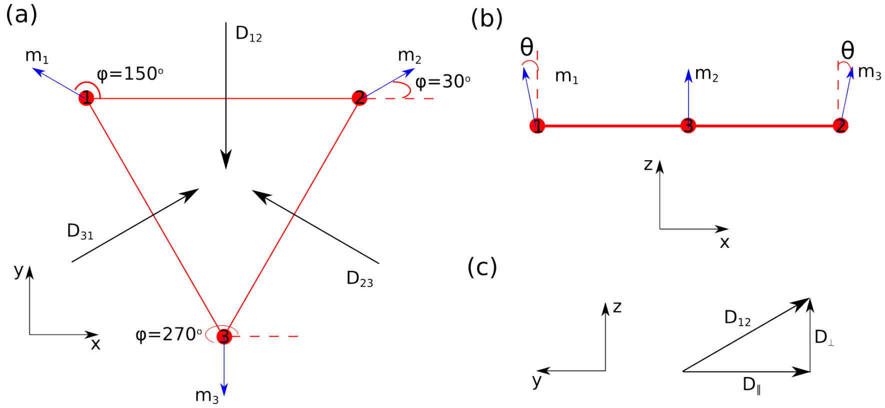

Supplementary Figure 3 | Nearly out-of-plane spin configuration. (a) and (b) respectively are top and side views of the Fe trimer in the nearly out-of-plane configuration. Red balls represent the adatoms with the label on top of the balls. Black arrows represent the DM vectors, while blue arrows represent the local magnetic moments (the in-plane projections of the magnetic moments have been enlarged for clarity). This spin structure respects the C_{3v} symmetry of the system. (c) Definition of the parallel and perpendicular components of the DM vector.

magnetic moments pointing nearly in-plane (see Supplementary Figure 4) with a non-collinearity angle of approximately $x = 4^\circ$ (see Supplementary Figure 5).

The opening of the non-collinearity angles can be related to the exchange parameters by making use of a classical Heisenberg model:

$$H = +J \sum_{i<j} \hat{\mathbf{m}}_i \cdot \hat{\mathbf{m}}_j + \sum_{i<j} \mathbf{D}_{ij} \cdot (\hat{\mathbf{m}}_i \times \hat{\mathbf{m}}_j) + K \sum_i m_{i,z}^2 \equiv H_J + H_D + H_K, \tag{4}$$

where $\hat{\mathbf{m}}_i = (\cos\varphi_i \sin\theta_i, \sin\varphi_i \sin\theta, \cos\theta_i), \quad i = 1, 2, 3$ are the magnetization unit vectors of the adatoms arranged as in Supplementary Figures 3 and 4. Note that within the present convention $J < 0$ ($J > 0$) gives rise to a ferromagnetic (antiferromagnetic) coupling, and $K < 0$ ($K > 0$) favors out-of-plane (in-plane) orientation of the magnetic moments.

We now compute an analytic solution for the direction of the spin moments illustrated in Supplementary Figures 3 and 4. Let us begin with the nearly in-plane configuration and thus set the azimuthal angle of all adatoms to $\varphi_i = 0$, *i.e.* the direction of the spin-moments are

$$\hat{\mathbf{m}}_i = (\sin\theta_i, 0, \cos\theta_i), \quad i = 1, 2, 3. \tag{5}$$

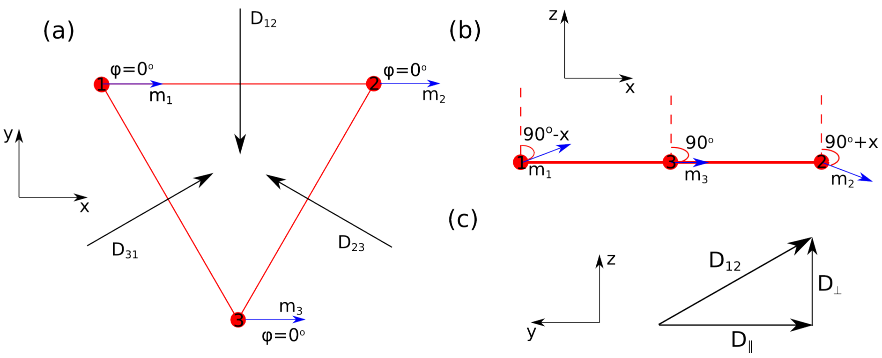

Supplementary Figure 4 | Nearly in-plane spin configuration. (a) and (b) respectively are top and side views of the Fe trimer in the nearly in-plane configuration. Labels are as in Supplementary Figure 3. We note that, although this spin structure breaks the C_{3v} symmetry of the system, there are two other solutions which are degenerate in energy with it and transform into each other according to the C_{3v} symmetry. (c) Definition of the parallel and perpendicular components of the DM vector.

The three contributions to the Hamiltonian of Eq. 4 are then given by:

$$H_J = J\sum_{i<j=1}^{3}\Big(\sin\theta_i\sin\theta_j+\cos\theta_i\cos\theta_j\Big), \tag{6}$$

$$H_D = -D_\parallel\Big(\cos\theta_1\sin\theta_2-\cos\theta_2\sin\theta_1\Big)$$
$$+\frac{1}{2}D_\parallel\Big(\cos\theta_2\sin\theta_3-\cos\theta_3\sin\theta_2\Big)+\frac{1}{2}D_\parallel\Big(\cos\theta_3\sin\theta_1-\cos\theta_1\sin\theta_3\Big), \tag{7}$$

$$H_K = K\Big(\cos^2\theta_1+\cos^2\theta_2+\cos^2\theta_3\Big). \tag{8}$$

We seek next for solutions near $\theta_i=\pi/2$ by using $\sin\theta_i=\sin(\pi/2+x_i)=\cos x_i\sim 1-x_i^2/2$, $\cos\theta_i=\cos(\pi/2+x_i)=-\sin x_i\sim -x_i$, allowing to write the full Hamiltonian as

$$H = J\Big(x_1x_2+x_1x_3+x_2x_3\Big)+K\Big(x_1^2+x_2^2+x_3^2\Big)+\frac{3}{2}D_\parallel\Big(x_1-x_2\Big)+3J. \tag{9}$$

The x_i that minimize this Hamiltonian are obtained from the solution of three coupled equations $\partial H/\partial x_i=0$, yielding

$$\begin{aligned} x_1 &= +\frac{3}{2}\frac{D_\parallel}{J-2K},\\ x_2 &= -\frac{3}{2}\frac{D_\parallel}{J-2K},\\ x_3 &= 0. \end{aligned} \tag{10}$$

The corresponding total energy for the nearly in-plane configuration is then given by

$$E_{\text{in}} \equiv \frac{9D_\parallel^2}{4J-8K}+3J\sim\frac{5D_\parallel^2}{4J}+3J. \tag{11}$$

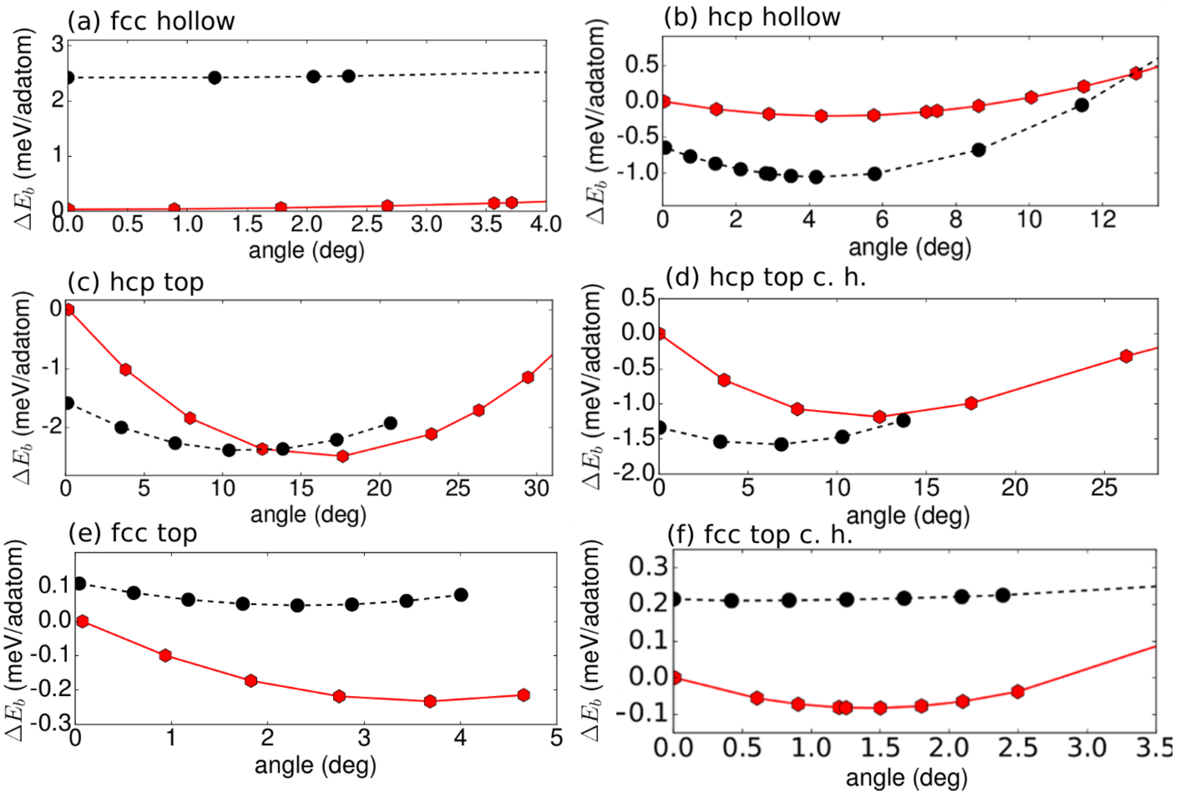

Supplementary Figure 5 | Non-collinear band calculations. DFT non-collinear band-energy calculations following the magnetic force theorem. The points in red denote the out-of-plane case, the black points the in-plane case, respectively. Solid and dashed lines are guides to the eye.

Given that J is negative (ferromagnetic coupling), this energy is also negative.

We next turn to the nearly out-of-plane configuration illustrated in Supplementary Figure 3 by fixing the azimuthal angles $\varphi_1 = 30°$, $\varphi_2 = 150°$ and $\varphi_3 = 270°$ and setting the same polar angle for the three adatoms:

$$\hat{\mathbf{m}}_1 = (-\frac{\sqrt{3}}{2}\sin\theta, \frac{1}{2}\sin\theta, \cos\theta), \tag{12}$$

$$\hat{\mathbf{m}}_2 = (\frac{\sqrt{3}}{2}\sin\theta, \frac{1}{2}\sin\theta, \cos\theta), \tag{13}$$

$$\hat{\mathbf{m}}_3 = (0, -\sin\theta, \cos\theta). \tag{14}$$

Then, the three parts of the Hamiltonian can be expressed as (after straightforward algebra in the J and DM parts):

$$H_J = 3J\Big(-\frac{1}{2}\sin^2\theta + \cos^2\theta\Big), \tag{15}$$

$$H_D = 3\sqrt{3}D_{\|}\sin\theta\cos\theta - \frac{3\sqrt{3}}{2}D_{\perp}\sin^2\theta, \tag{16}$$

$$H_K = 3K\cos^2\theta. \tag{17}$$

We next assume small deviations $\sin\theta \sim \theta$, $\cos\theta \sim 1-\theta^2/2$, allowing to write the full Hamiltonian as

$$H = -\frac{3}{2}J\theta^2 + 3\sqrt{3}D_{\|}x - \frac{3\sqrt{3}}{2}D_{\perp}\theta^2 - 3K\theta^2 + 3K + 3J. \tag{18}$$

The solution of $\partial H/\partial\theta = 0$ is

$$\theta = \frac{\sqrt{3}D_{\|}}{J + 2K + \sqrt{3}D_{\perp}}. \tag{19}$$

In all cluster types studied in this work the hierarchy $|J| \gg |D_{\|}|, |D_{\perp}|, |K|$ is fulfilled. Therefore, the angles predicted by Eqs. 10 and 19 reveal a larger impact of non-collinearity on the nearly out-of-plane configuration than in the nearly in-plane configuration by a factor of approximately $2/\sqrt{3} \sim 1.15$, which is in reasonable accordance with the *ab initio* band calculations shown in Supplementary Figure 5.

Inserting Eq. 19 into Eq. 18 one obtains the total minimum energy of the nearly out-of-plane configuration,

$$E_{\text{out}} = 3(K + J) + \frac{9}{2}\frac{D_{\|}^2}{\sqrt{3}D_{\perp} + J + 2K} \sim \frac{9D_{\|}^2}{2J} + 3K + 3J. \tag{20}$$

Thus, the energy difference between Eq. 11 and Eq. 20 gives the relative energy gain induced by non-collinearity between the nearly in-plane and out-of-plane configurations:

$$E_{\text{out}} - E_{\text{in}} \sim \frac{9D_{\|}^2}{4J} + 3K. \tag{21}$$

The above equation shows that, in absence of MAE ($K \sim 0$), $E_{\text{out}} - E_{\text{in}} < 0$ as long as the coupling is ferromagnetic, *i.e.* the nearly out-of-plane direction is favoured by non-collinearity. Interestingly, even if $K > 0$ (MAE favours in-plane orientation), the overal total energy may still favour the nearly out-of-plane configuration provided $\left|9D_{\|}^2/4J\right| > |3K|$ is fulfilled.

Supplementary Note 3 | Fano functions

In order to extract the magnetic field dependent shift and temperature induced broadening of the Kondo resonance (Fig.3 of the main manuscript), we fitted the measured voltage dependent spectra $\frac{\mathrm{d}I}{\mathrm{d}V}(V)$ to the following sum of two Fano functions [10] including a linear background:

$$G(V) = G_1 \cdot \frac{(q+\varepsilon_1)^2}{1+\varepsilon_1^2} + G_2 \cdot \frac{(q+\varepsilon_2)^2}{1+\varepsilon_2^2} + G_{\text{off}} + G_{\text{lin}} \cdot V \tag{22}$$

$$\varepsilon_i = \frac{eV - E_i}{\mathit{\Gamma}} \tag{23}$$

Here, q is the so-called form factor, $\mathit{\Gamma}$ is the full width at half maximum and E_i are the energetic positions of the Kondo resonances. The parameters extracted by fitting such functions to the experimental data are given in Supplementary Tables 3 and 4, and the two fitted Fano functions for the case of the magnetic field dependence are shown in Supplementary Fig.6. Note, that the resulting q-factors are negative, indicating strong phase shifts between the two tunneling paths [11].

Supplementary Table 3 | Parameters for magnetic field dependent Fano functions. Parameters used for the fits of the B dependent spectra of the hcp-hollow cluster (Fig.3a of main manuscript).

B (T)	$\mathit{\Gamma}$ (meV)	E_1 (meV)	E_2 (meV)	q	G_1 (a.u.)	G_2 (a.u.)
0	0.32	-0.05	-	-1.60	1.68	-
1.5	0.42	0.15	-0.14	-1.52	1.99	1.44
3	0.34	0.25	-0.43	-2.98	0.73	0.49
4.5	0.37	0.31	-0.60	-5.01	0.31	0.18
6	0.53	0.49	-0.59	-3.08	0.79	0.26
7.5	0.73	0.69	-0.80	-2.30	1.36	0.19

Supplementary Table 4 | Parameters for temperature dependent Fano functions. Parameters used for the fits of the T dependent spectra measured on the hcp-hollow cluster (Fig.3b of main manuscript).

T (K)	$\mathit{\Gamma}$ (meV)	E_1 (meV)	q	G_1 (a.u.)
0.31	0.40	0.054	-0.78	5.3
1.3	0.70	0.062	-0.81	4.3
2.3	1.30	0.031	-0.89	3.9
3.1	1.62	0.022	-0.80	4.1
4.3	2.13	-0.034	-1.04	3.3
5.3	2.82	-0.126	-1.04	3.8
6.2	3.10	0.058	-1.22	2.7

In order to estimate the Kondo temperature T_{K}, the resulting temperature dependence of Γ was fitted to the power law [12] $\Gamma(T) = \frac{1}{2}\sqrt{(\alpha k_{\mathrm{B}}T)^2 + (2k_{\mathrm{B}}T_{\mathrm{K}})^2}$ resulting in $T_{\mathrm{K}} = 4.19\,\mathrm{K}$ ($\alpha = 11.8$) which is illustrated as a grey line in Fig.3d together with the data. Additionally, we compare to published numerical renormalization group calculations for a spin-1/2 impurity in the strong coupling regime [13], given by the grey dots in Fig.3d, resulting in a similar value of $T_{\mathrm{K}} = 4.64\,\mathrm{K}$.

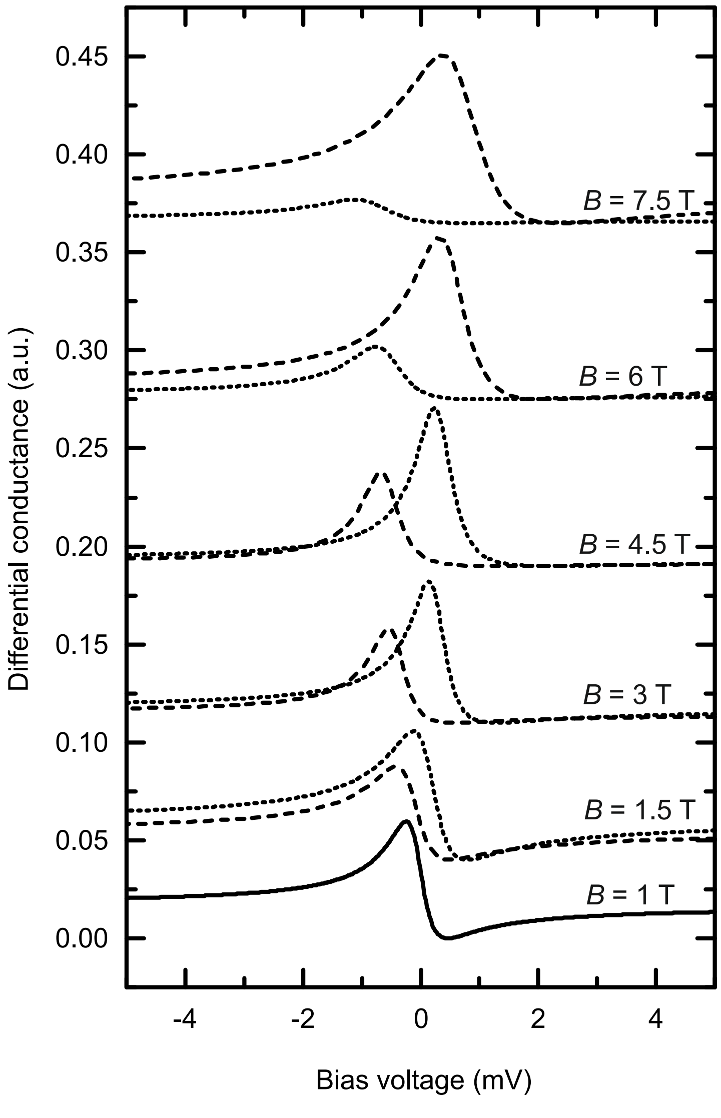

Supplementary Figure 6 | Fano functions. Here, the used Fano functions for the different magnetic fields as indicated are plotted without the linear background ($G_{\mathrm{lin}} = 0$) but with artificial offsets for better visibility.

Supplementary Note 4 | Inelastic scanning tunneling spectroscopy

Supplementary Fig.7 (left panels) show $\mathrm{d}I/\mathrm{d}V$ curves measured on the hcp top, fcc hollow and fcc top GMCs as a function of the out-of-plane oriented magnetic field. They reveal features at positive and negative bias voltage linearly increasing symmetrically around the Fermi energy ($V = 0\,\mathrm{mV}$) as a function of B. The associated $\mathrm{d}^2I/\mathrm{d}V^2$ curves (right panels) have peaks and dips at the corresponding bias voltages. This behavior is the fingerprint of a spin excitation of an out-of-plane easy axis system [1]. Note, however, that the shape of the features in the spin excitation spectra partly deviates from the usual step-like appearance typically found for magnetic atoms which are more strongly decoupled from a metallic substrate [14,15]. This might indicate deviations from the simple effective-spin model where the magnetic impurity is artificially separated into an interior part (effective spin) that is excited by the tunneling electrons, and an exterior part that interacts with the effective spin leading to damping of the spin excitations. In the present system, this border is not well defined due to the GMC character of the magnetic impurity, which can lead to excitation spectra that strongly deviate from the simple step shape [16]. We extract the corresponding excitation energies $\mathit{\Delta}_{01}$ by searching the center of the symmetric peaks/dips in the $\mathrm{d}^2I/\mathrm{d}V^2$ curves, which are marked by crosses, both in the $\mathrm{d}I/\mathrm{d}V$ as well as in the $\mathrm{d}^2I/\mathrm{d}V^2$ curves. The extracted excitation energies $\mathit{\Delta}_{01}$ are plotted in Supplementary Fig.8 as a function of B. Indeed, there is a linear behavior, as expected from the out-of-plane system. By fitting a linear function to these plots, we extracted the g-factors via $g = 1/\mu_\mathrm{B} \cdot \mathrm{d}\mathit{\Delta}_{01}(B)/\mathrm{d}B$. The accordingly determined parameters $\mathit{\Delta}_{01}$ and g of the three GMCs are given in Supplementary Table 5. Here, we distinguish between the values determined from the excitation on the negative (neg.) and positive (pos.) bias voltage sides, which allows us to estimate the error, which is given in the Table of the main manuscript text.

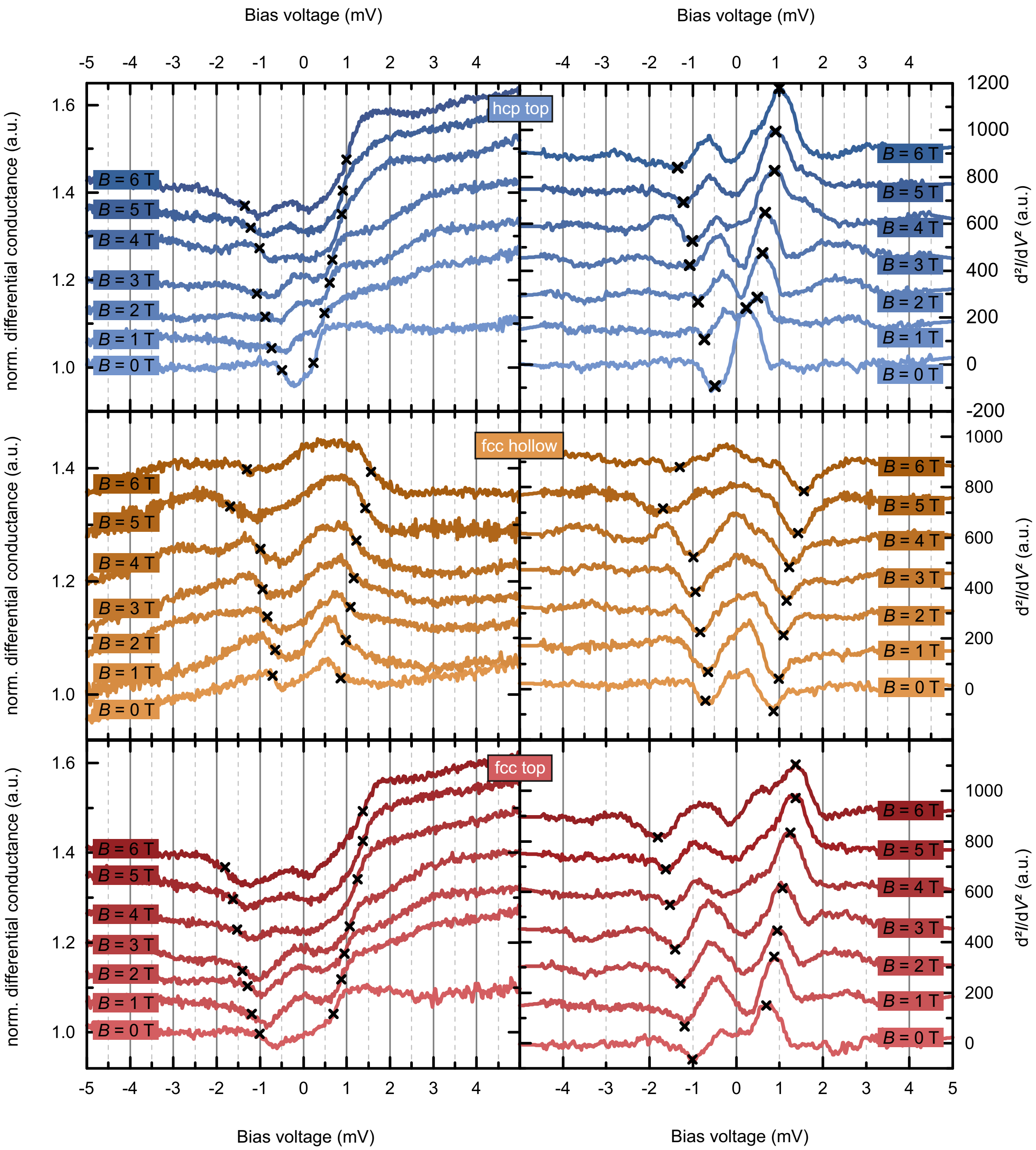

Supplementary Figure 7 | Inelastic scanning tunneling spectroscopy of hcp top, fcc hollow and fcc top clusters. $\mathrm{d}I/\mathrm{d}V$ (left panels) and $\mathrm{d}^2I/\mathrm{d}V^2$ (right panels) as calculated by smoothing (every datapoint is the average of the 50 surrounding datapoints) and consecutive numerical differentiation of the $\mathrm{d}I/\mathrm{d}V$ spectra that have been measured by Lock-In technique. The crosses mark the positions of the extracted spin-excitation energies Δ_{01}. Tunneling parameters: $V_{\mathrm{stab}} = 5\,\mathrm{mV}$, $I_{\mathrm{stab}} = 2\,\mathrm{nA}$, $V_{\mathrm{mod}} = 80\,\mu\mathrm{V}$, $T = 0.3\,\mathrm{K}$.

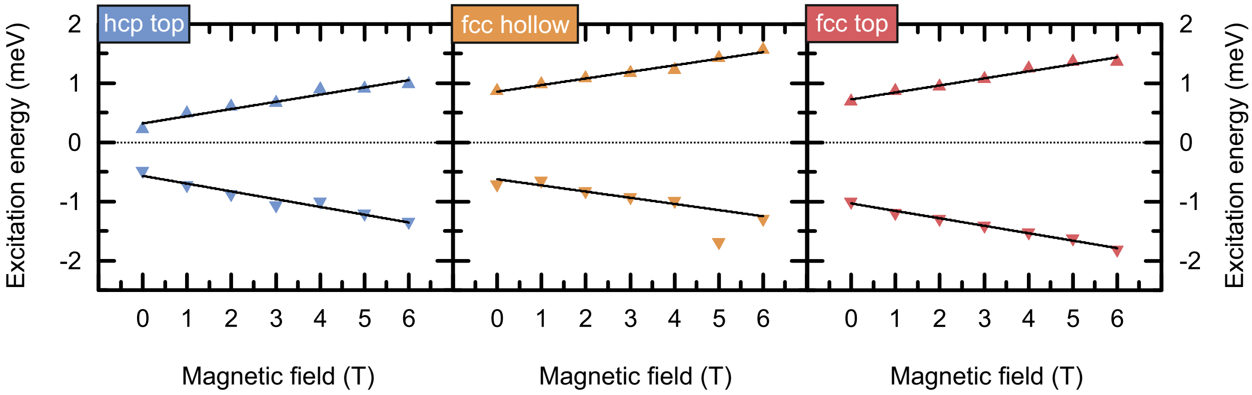

Supplementary Figure 8 | Spin excitation energies determined from inelastic scanning tunneling spectroscopy. The plotted spin excitation energies Δ_{01} (markers) were determined from the spectra in Supplementary Fig.7. The lines are fits of the excitation energies on the positive and negative side to linear functions.

Supplementary Table 5 | Zero field excitation energies Δ_{01} and g-factors. The values were determined from the zero field excitation energies and fitted lines on the negative (neg.) and positive (pos.) energy side in Supplementary Fig.8.

cluster type	Δ_{01} (meV, neg.)	Δ_{01} (meV, pos.)	g (neg.)	g (pos.)
hcp top	-0.57	0.32	2.26	2.11
fcc hollow	-0.62	0.86	1.8	1.92
fcc top	-1.03	0.73	2.18	2.06

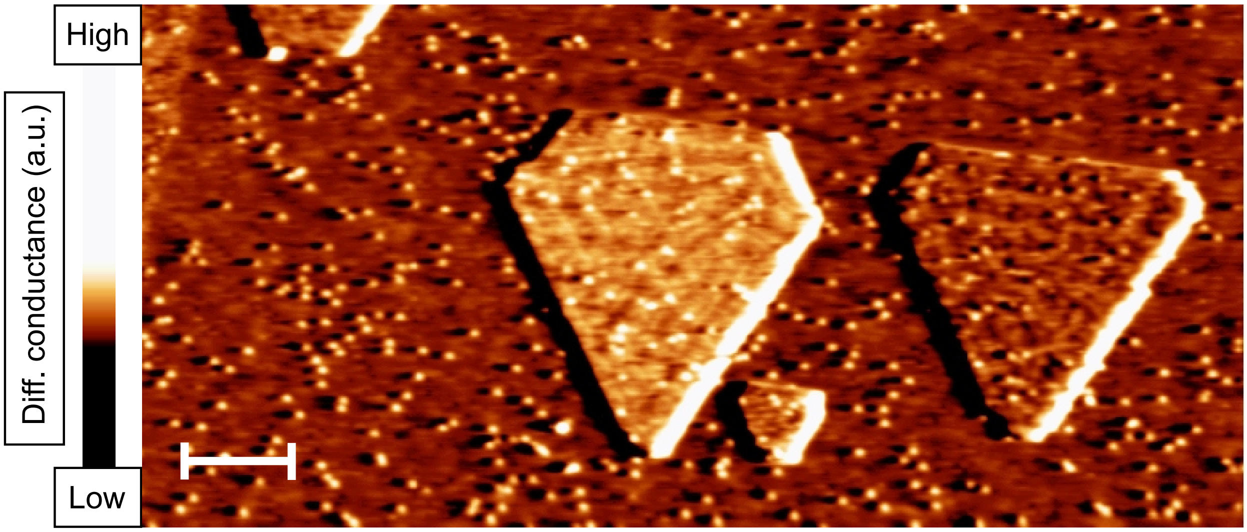

Supplementary Figure 9 | Spin-resolved differential conductance image of Cobalt islands on Pt(111). Differential conductance image of the sample with triangular shaped Co monolayer islands and Fe atoms on Pt(111). The large left (right) islands appears bright (dark) indicating their opposite out-of-plane magnetizations and the sensitivity of the tip to the out-of-plane component of the sample magnetization. The scale bar defines a length of $10\,\mathrm{nm}$. Tunneling parameters: $V = -10\,\mathrm{mV}$, $I = 750\,\mathrm{pA}$, $V_{\mathrm{mod}} = 5\,\mathrm{mV}$, $B = 0\,\mathrm{T}$, $T = 0.3\,\mathrm{K}$.

Supplementary References

1. Khajetoorians, A. A. *et al.* Spin excitations of individual Fe atoms on Pt(111): Impact of the site-dependent giant substrate polarization. *Phys. Rev. Lett.* **111**, 157204 (2013).

2. Papanikolaou, N., Zeller, R. & Dederichs, P. H. Conceptual improvements of the KKR method. *Journal of Physics: Condensed Matter* **14**, 2799 (2002).

3. dos Santos Dias, M., Schweflinghaus, B., Blügel, S. & Lounis, S. Relativistic dynamical spin excitations of magnetic adatoms. *Physical Review B* **91**, 075405 (2015).

4. Vosko, S. H., Wilk, L. & Nusair, M. Accurate spin-dependent electron liquid correlation energies for local spin density calculations: a critical analysis. *Canadian Journal of Physics* **58**, 1200–1211 (1980).

5. Giannozzi, P. *et al.* QUANTUM ESPRESSO: a modular and open-source software project for quantum simulations of materials. *Journal of Physics: Condensed Matter* **21**, 395502 (2009).

6. Weinert, M., Watson, R. E. & Davenport, J. W. Total-energy differences and eigenvalue sums. *Physical Review B* **32**, 2115–2119 (1985).

7. Liechtenstein, A. I., Katsnelson, M. I. & Gubanov, V. A. Exchange interactions and spin-wave stiffness in ferromagnetic metals. *Journal of Physics F: Metal Physics* **14**, L125 (1984).

8. Polesya, S. *et al.* Finite-temperature magnetism of Fe_xPd_{1-x} and Co_xPt_{1-x} alloys. *Physical Review B* **82**, 214409 (2010).

9. Udvardi, L., Szunyogh, L., Palotás, K. & Weinberger, P. First-principles relativistic study of spin waves in thin magnetic films. *Physical Review B* **68**, 104436 (2003).

10. Ternes, M., Heinrich, A. J. & Schneider, W.-D. Spectroscopic manifestations of the Kondo effect on single adatoms. *Journal of Physics: Condensed Matter* **21**, 053001 (2009).

11. Prüser, H. *et al.* Long-range Kondo signature of a single magnetic impurity. *Nat Phys* **7**, 203–206 (2011).

12. Khajetoorians, A. A. *et al.* Tuning emergent magnetism in a Hund's impurity. *Nature Nanotechnology* **10**, 958–964 (2015).

13. Costi, T. A. Kondo effect in a magnetic field and the magnetoresistivity of Kondo alloys. *Phys. Rev. Lett.* **85**, 1504–1507 (2000).

14. Hirjibehedin, C. F. *et al.* Large magnetic anisotropy of a single atomic spin embedded in a surface molecular network. *Science* **317**, 1199–1203 (2007).

15. Khajetoorians, A. A. *et al.* Detecting excitation and magnetization of individual dopants in a semiconductor. *Nature* **467**, 1084–1087 (2010).

16. Schweflinghaus, B., dos Santos Dias, M. & Lounis, S. Observing spin excitations in $3d$ transition-metal adatoms on pt(111) with inelastic scanning tunneling spectroscopy: A first-principles perspective. *Phys. Rev. B* **93**, 035451 (2016).
